# Diaqua­bis(1,10-phenanthroline)magnesium dichromate(VI) 1,10-phenanthroline disolvate

**DOI:** 10.1107/S1600536809030128

**Published:** 2009-08-08

**Authors:** Hai-Xing Liu, Gui-Ying Dong, Zhi-Hong Ma, Guang-Hua Cui

**Affiliations:** a Microscale Science Institute, College of Chemistry and Chemical Engineering, Weifang University, Weifang 261061, People’s Republic of China; b College of Chemical Engineering and Biotechnology, Hebei Polytechnic University, Tangshan 063009, People’s Republic of China; c College of Basic Medicine, Hebei Medical University, Shijiazhuang 050017, People’s Republic of China

## Abstract

In the title compound, [Mg(C_12_H_8_N_2_)_2_(H_2_O)_2_][Cr_2_O_7_]·2C_12_H_8_N_2_, the cation and anion are situated on a twofold rotation axis. The Mg^II^ ion is coordinated by four N atoms from two 1,10-phenanthroline ligands and two O atoms from coordinated water mol­ecules in a distorted octa­hedral geometry. Inter­molecular O—H⋯N and O—H⋯O hydrogen bonds and π–π inter­actions between the aromatic rings [shortest centroid–centroid separation = 3.527 (2) Å] link the cations, anions and 1,10-phenanthroline solvent mol­ecules into a hydrogen-bonded cluster.

## Related literature

For related magnesium–phenanthroline complexes, see: Zhu *et al.* (2008 ▶); Hao *et al.* (2008 ▶); Zhang (2004 ▶).
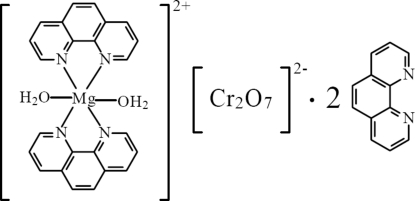


## Experimental

### 

#### Crystal data

[Mg(C_12_H_8_N_2_)_2_(H_2_O)_2_][Cr_2_O_7_]·2C_12_H_8_N_2_
*M* *_r_* = 997.16
Monoclinic, 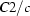

*a* = 16.761 (3) Å
*b* = 22.172 (4) Å
*c* = 13.996 (3) Å
β = 123.49 (3)°
*V* = 4338 (2) Å^3^
*Z* = 4
Mo *K*α radiation
μ = 0.59 mm^−1^
*T* = 293 K
0.30 × 0.28 × 0.21 mm

#### Data collection

Bruker SMART CCD area-detector diffractometer
Absorption correction: multi-scan (*SADABS*; Sheldrick, 1996 ▶) *T*_min_ = 0.826, *T*_max_ = 0.878
16762 measured reflections
3817 independent reflections
3068 reflections with *I* > 2σ(*I*)
*R*_int_ = 0.020

#### Refinement

*R*[*F* ^2^ > 2σ(*F* ^2^)] = 0.051
*wR*(*F* ^2^) = 0.155
*S* = 1.06
3817 reflections
308 parameters
H-atom parameters constrained
Δρ_max_ = 0.87 e Å^−3^
Δρ_min_ = −0.46 e Å^−3^

### 

Data collection: *SMART* (Bruker, 1998 ▶); cell refinement: *SAINT* (Bruker, 1999 ▶); data reduction: *SAINT*; program(s) used to solve structure: *SHELXS97* (Sheldrick, 2008 ▶); program(s) used to refine structure: *SHELXL97* (Sheldrick, 2008 ▶); molecular graphics: *SHELXTL* (Sheldrick, 2008 ▶); software used to prepare material for publication: *SHELXTL*.

## Supplementary Material

Crystal structure: contains datablocks I, global. DOI: 10.1107/S1600536809030128/cv2597sup1.cif

Structure factors: contains datablocks I. DOI: 10.1107/S1600536809030128/cv2597Isup2.hkl

Additional supplementary materials:  crystallographic information; 3D view; checkCIF report

## Figures and Tables

**Table 1:** Hydrogen-bond geometry (Å, °)

*D*—H⋯*A*	*D*—H	H⋯*A*	*D*⋯*A*	*D*—H⋯*A*
O1*W*—H1*A*⋯N3	0.85	2.08	2.876 (4)	156
O1*W*—H1*B*⋯O1	0.85	1.84	2.636 (4)	154

**Fig. 1.**
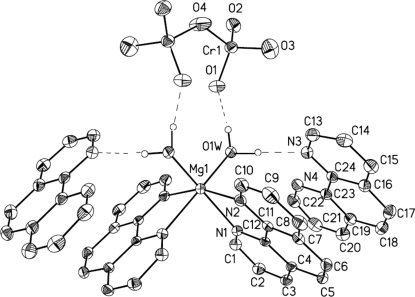
The hydrogen-bonded (dashed lines) cluster in (I) showing the atomic numbering and 30% probability displacement ellipsoids. Unlabelled atoms are related with the labelled ones by symmetry [-x + 1, y, 1/2 - z]. H atoms not involved in hydrogen-bonding are omitted for clarity.

## supplementary crystallographic information

### Comment

1,10-Phenanthroline (phen), which is the parent of an important class of chelating agents, has been widely used in the construction of supramolecular architectures. Some magnesium(II)-phenanthroline complexes have been synthesized and reported (Zhu *et al.*, 2008; Hao *et al.* 2008; Zhang, 2004). As a continuation of these studies, we now report the crystal structure of the title complex (I).

X-ray structure analysis reveals that (I) is an ionic monomeric Mg^II^ complex (Fig. 1) with two solvent phen molecules. The Mg(II) ion is surrounded by four N atoms from the two phen ligands and two O atoms from two coordinated water molecules to form distorted MgN_4_O_2_ octahedron. The Mg—O(2.018 (2)–2.018 (3) Å) and Mg—N (2.211 (3)–2.215 (2) Å) bond lengths are normal.

In the crystal structure, intermolecular O—H···N and O—H···O hydrogen bonds (Table 1) and π–π interactions between the aromatic rings with the shortest centroid-centroid separation of 3.527 (2) Å, link cation, anion and two solvent 1,10-phenanthroline molecules into a hydrogen-bonded cluster (Fig. 1).

### Experimental

Magnesium chloride, potassium dichromate and 1,10-phenanthroline (molar ratio 1:1:4) were dissolved in water-ethanol mixture (1:1 *v*/*v*, 50 ml) and refluxed for 3 h. The resulting solution was allowed to stand at room temperature for a week and yellow crystals of (I) were obtained.

### Refinement

C-bound H atoms were geometrically positioned [O—H = 0.93 Å], while water H atoms were located in a Fourier difference map, but placed in idealized positions [O—H = 0.85 Å]. All H atoms were  refined as riding, with *U*_iso_(H) =1.2*U*_eq_(C, O).

### Crystal data

**Table:**

[Mg(C_12_H_8_N_2_)_2_(H_2_O)_2_][Cr_2_O_7_]·2C_12_H_8_N_2_	*F*(000) = 2048
*M**_r_* = 997.16	*D*_x_ = 1.527 Mg m^−^^3^
Monoclinic, *C*2/*c*	Mo *K*α radiation, λ = 0.71073 Å
Hall symbol: -C 2yc	Cell parameters from 2800 reflections
*a* = 16.761 (3) Å	θ = 5.0–22.8°
*b* = 22.172 (4) Å	µ = 0.59 mm^−^^1^
*c* = 13.996 (3) Å	*T* = 293 K
β = 123.49 (3)°	Prism, yellow
*V* = 4338 (2) Å^3^	0.30 × 0.28 × 0.21 mm
*Z* = 4	

### Data collection

**Table:**

Bruker SMART CCD area-detector diffractometer	3817 independent reflections
Radiation source: fine-focus sealed tube	3068 reflections with *I* > 2σ(*I*)
graphite	*R*_int_ = 0.020
φ and ω scans	θ_max_ = 25.0°, θ_min_ = 3.1°
Absorption correction: multi-scan (*SADABS*; Sheldrick, 1996)	*h* = −19→19
*T*_min_ = 0.826, *T*_max_ = 0.878	*k* = −26→26
16762 measured reflections	*l* = −16→15

### Refinement

**Table:**

Refinement on *F*^2^	Primary atom site location: structure-invariant direct methods
Least-squares matrix: full	Secondary atom site location: difference Fourier map
*R*[*F*^2^ > 2σ(*F*^2^)] = 0.051	Hydrogen site location: inferred from neighbouring sites
*wR*(*F*^2^) = 0.155	H-atom parameters constrained
*S* = 1.06	*w* = 1/[σ^2^(*F*_o_^2^) + (0.0878*P*)^2^ + 5.6611*P*] where *P* = (*F*_o_^2^ + 2*F*_c_^2^)/3
3817 reflections	(Δ/σ)_max_ < 0.001
308 parameters	Δρ_max_ = 0.87 e Å^−^^3^
0 restraints	Δρ_min_ = −0.46 e Å^−^^3^

### Special details

**Table:**

Geometry. All e.s.d.'s (except the e.s.d. in the dihedral angle between two l.s. planes) are estimated using the full covariance matrix. The cell e.s.d.'s are taken into account individually in the estimation of e.s.d.'s in distances, angles and torsion angles; correlations between e.s.d.'s in cell parameters are only used when they are defined by crystal symmetry. An approximate (isotropic) treatment of cell e.s.d.'s is used for estimating e.s.d.'s involving l.s. planes.
Refinement. Refinement of *F*^2^ against ALL reflections. The weighted *R*-factor *wR* and goodness of fit *S* are based on *F*^2^, conventional *R*-factors *R* are based on *F*, with *F* set to zero for negative *F*^2^. The threshold expression of *F*^2^ > σ(*F*^2^) is used only for calculating *R*-factors(gt) *etc*. and is not relevant to the choice of reflections for refinement. *R*-factors based on *F*^2^ are statistically about twice as large as those based on *F*, and *R*- factors based on ALL data will be even larger.

### Fractional atomic coordinates and isotropic or equivalent isotropic displacement parameters (Å^2^)

**Table:**

	*x*	*y*	*z*	*U*_iso_*/*U*_eq_	
O4	0.5000	0.0547 (2)	0.2500	0.135 (2)	
Mg1	0.5000	0.32609 (6)	0.2500	0.0429 (3)	
C12	0.42272 (18)	0.41277 (11)	0.3486 (2)	0.0341 (6)	
C4	0.36810 (18)	0.45551 (13)	0.3620 (2)	0.0387 (6)	
N1	0.40575 (15)	0.39775 (10)	0.24512 (19)	0.0374 (5)	
N2	0.55208 (16)	0.34161 (11)	0.4315 (2)	0.0427 (6)	
C7	0.5222 (2)	0.39865 (14)	0.5573 (2)	0.0431 (7)	
C11	0.50124 (18)	0.38299 (12)	0.4484 (2)	0.0367 (6)	
O1W	0.60296 (19)	0.26585 (11)	0.2850 (2)	0.0743 (8)	
C5	0.3893 (2)	0.46843 (14)	0.4739 (3)	0.0479 (7)	
H5	0.3515	0.4957	0.4826	0.057*	
C2	0.2783 (2)	0.46981 (15)	0.1593 (3)	0.0503 (7)	
H2	0.2303	0.4890	0.0930	0.060*	
C3	0.2941 (2)	0.48458 (14)	0.2624 (3)	0.0471 (7)	
H3	0.2565	0.5136	0.2674	0.057*	
C6	0.4639 (2)	0.44126 (15)	0.5673 (3)	0.0509 (8)	
H6	0.4772	0.4507	0.6395	0.061*	
C10	0.6264 (2)	0.31578 (16)	0.5247 (3)	0.0557 (8)	
H10	0.6616	0.2871	0.5145	0.067*	
C1	0.3345 (2)	0.42583 (14)	0.1536 (3)	0.0469 (7)	
H1	0.3217	0.4155	0.0820	0.056*	
C9	0.6539 (2)	0.32924 (18)	0.6350 (3)	0.0637 (10)	
H9	0.7068	0.3104	0.6971	0.076*	
C8	0.6026 (2)	0.37051 (17)	0.6519 (3)	0.0575 (9)	
H8	0.6205	0.3801	0.7260	0.069*	
H1B	0.5962	0.2278	0.2851	0.086*	
H1A	0.6369	0.2686	0.2572	0.086*	
C24	0.7163 (2)	0.30719 (14)	0.1147 (3)	0.0448 (7)	
C16	0.7005 (2)	0.32042 (15)	0.0067 (3)	0.0496 (7)	
C23	0.7918 (2)	0.33854 (14)	0.2156 (3)	0.0467 (7)	
C19	0.8456 (2)	0.38268 (16)	0.2027 (3)	0.0513 (8)	
N3	0.66308 (19)	0.26634 (13)	0.1282 (2)	0.0533 (7)	
N4	0.8060 (2)	0.32422 (14)	0.3182 (2)	0.0577 (7)	
C18	0.8266 (2)	0.39479 (17)	0.0912 (3)	0.0585 (9)	
H18	0.8621	0.4241	0.0830	0.070*	
C17	0.7583 (2)	0.36451 (18)	−0.0013 (3)	0.0590 (9)	
H17	0.7486	0.3725	−0.0722	0.071*	
C13	0.5949 (3)	0.23787 (17)	0.0359 (3)	0.0611 (9)	
H13	0.5584	0.2097	0.0448	0.073*	
C14	0.5743 (3)	0.24734 (17)	−0.0736 (3)	0.0641 (9)	
H14	0.5252	0.2262	−0.1358	0.077*	
C20	0.9162 (2)	0.41347 (18)	0.3012 (3)	0.0642 (10)	
H20	0.9527	0.4434	0.2963	0.077*	
C15	0.6273 (2)	0.28822 (17)	−0.0877 (3)	0.0601 (9)	
H15	0.6151	0.2949	−0.1602	0.072*	
C21	0.9307 (3)	0.3991 (2)	0.4040 (3)	0.0712 (11)	
H21	0.9775	0.4188	0.4704	0.085*	
C22	0.8749 (3)	0.35454 (19)	0.4088 (3)	0.0681 (10)	
H22	0.8863	0.3452	0.4801	0.082*	
Cr1	0.60986 (4)	0.08803 (3)	0.29232 (5)	0.0543 (2)	
O2	0.69117 (19)	0.04355 (13)	0.3843 (2)	0.0739 (8)	
O3	0.6203 (3)	0.09376 (18)	0.1873 (2)	0.1033 (11)	
O1	0.6225 (2)	0.15268 (13)	0.3521 (3)	0.0874 (9)	

### Atomic displacement parameters (Å^2^)

**Table:**

	*U*^11^	*U*^22^	*U*^33^	*U*^12^	*U*^13^	*U*^23^
O4	0.069 (3)	0.058 (3)	0.271 (8)	0.000	0.090 (4)	0.000
Mg1	0.0517 (8)	0.0367 (8)	0.0523 (8)	0.000	0.0364 (7)	0.000
C12	0.0324 (13)	0.0281 (14)	0.0418 (14)	−0.0044 (10)	0.0205 (11)	−0.0012 (10)
C4	0.0358 (13)	0.0335 (15)	0.0485 (16)	−0.0045 (11)	0.0243 (13)	−0.0040 (11)
N1	0.0372 (12)	0.0352 (13)	0.0380 (12)	0.0007 (9)	0.0196 (10)	−0.0003 (9)
N2	0.0403 (12)	0.0390 (14)	0.0523 (14)	0.0048 (10)	0.0277 (11)	0.0096 (10)
C7	0.0438 (15)	0.0441 (17)	0.0387 (14)	−0.0092 (12)	0.0211 (13)	0.0024 (12)
C11	0.0356 (13)	0.0338 (14)	0.0411 (14)	−0.0048 (11)	0.0214 (12)	0.0042 (11)
O1W	0.105 (2)	0.0451 (14)	0.125 (2)	0.0226 (13)	0.0963 (19)	0.0224 (14)
C5	0.0520 (16)	0.0456 (18)	0.0575 (18)	−0.0037 (13)	0.0375 (15)	−0.0070 (14)
C2	0.0419 (15)	0.0481 (18)	0.0508 (17)	0.0093 (13)	0.0192 (14)	0.0080 (14)
C3	0.0395 (14)	0.0388 (17)	0.0640 (19)	0.0052 (12)	0.0292 (14)	0.0006 (13)
C6	0.0643 (19)	0.0529 (19)	0.0462 (16)	−0.0120 (15)	0.0373 (16)	−0.0081 (14)
C10	0.0470 (17)	0.054 (2)	0.064 (2)	0.0127 (14)	0.0293 (16)	0.0199 (15)
C1	0.0470 (16)	0.0472 (18)	0.0414 (15)	0.0003 (13)	0.0213 (14)	0.0005 (13)
C9	0.0467 (17)	0.071 (3)	0.0535 (19)	0.0029 (16)	0.0154 (16)	0.0253 (17)
C8	0.0597 (19)	0.061 (2)	0.0399 (16)	−0.0079 (16)	0.0198 (15)	0.0083 (14)
C24	0.0423 (15)	0.0439 (17)	0.0507 (16)	0.0145 (12)	0.0273 (14)	0.0063 (13)
C16	0.0462 (16)	0.0527 (19)	0.0503 (17)	0.0174 (14)	0.0268 (14)	0.0135 (14)
C23	0.0446 (16)	0.0453 (18)	0.0514 (16)	0.0152 (13)	0.0273 (14)	0.0078 (13)
C19	0.0370 (15)	0.055 (2)	0.0565 (18)	0.0120 (13)	0.0227 (14)	0.0077 (15)
N3	0.0573 (15)	0.0498 (16)	0.0617 (16)	0.0011 (12)	0.0384 (14)	0.0005 (12)
N4	0.0641 (17)	0.0592 (18)	0.0527 (15)	0.0123 (14)	0.0341 (14)	0.0042 (13)
C18	0.0449 (17)	0.064 (2)	0.068 (2)	0.0101 (15)	0.0322 (17)	0.0191 (17)
C17	0.0526 (18)	0.069 (2)	0.0556 (19)	0.0134 (16)	0.0302 (17)	0.0215 (17)
C13	0.062 (2)	0.051 (2)	0.075 (2)	−0.0018 (16)	0.0403 (19)	−0.0026 (17)
C14	0.060 (2)	0.055 (2)	0.064 (2)	0.0039 (16)	0.0255 (18)	−0.0025 (16)
C20	0.0405 (17)	0.066 (2)	0.074 (2)	0.0054 (15)	0.0244 (17)	0.0047 (18)
C15	0.060 (2)	0.062 (2)	0.0485 (17)	0.0138 (17)	0.0241 (16)	0.0055 (16)
C21	0.049 (2)	0.079 (3)	0.062 (2)	0.0107 (18)	0.0160 (18)	−0.0087 (19)
C22	0.071 (2)	0.075 (3)	0.055 (2)	0.015 (2)	0.0322 (19)	0.0016 (18)
Cr1	0.0481 (3)	0.0484 (4)	0.0652 (4)	0.0060 (2)	0.0305 (3)	0.0057 (2)
O2	0.0757 (17)	0.0743 (19)	0.0658 (15)	0.0237 (14)	0.0353 (14)	0.0140 (13)
O3	0.106 (3)	0.132 (3)	0.0579 (16)	0.020 (2)	0.0364 (17)	0.0151 (17)
O1	0.113 (2)	0.0484 (16)	0.114 (2)	−0.0003 (15)	0.071 (2)	0.0028 (15)

### Geometric parameters (Å, °)

**Table:**

O4—Cr1	1.756 (2)	C9—C8	1.363 (5)
O4—Cr1^i^	1.756 (2)	C9—H9	0.9300
Mg1—O1W^i^	2.017 (2)	C8—H8	0.9300
Mg1—O1W	2.017 (2)	C24—N3	1.355 (4)
Mg1—N2	2.210 (3)	C24—C16	1.414 (4)
Mg1—N2^i^	2.210 (3)	C24—C23	1.451 (4)
Mg1—N1	2.215 (2)	C16—C15	1.404 (5)
Mg1—N1^i^	2.215 (2)	C16—C17	1.424 (5)
C12—N1	1.354 (3)	C23—N4	1.357 (4)
C12—C4	1.401 (4)	C23—C19	1.408 (5)
C12—C11	1.446 (4)	C19—C20	1.403 (5)
C4—C3	1.410 (4)	C19—C18	1.434 (5)
C4—C5	1.431 (4)	N3—C13	1.321 (4)
N1—C1	1.329 (4)	N4—C22	1.333 (5)
N2—C10	1.337 (4)	C18—C17	1.342 (5)
N2—C11	1.359 (4)	C18—H18	0.9300
C7—C11	1.406 (4)	C17—H17	0.9300
C7—C8	1.410 (4)	C13—C14	1.387 (5)
C7—C6	1.421 (5)	C13—H13	0.9300
O1W—H1B	0.8517	C14—C15	1.359 (5)
O1W—H1A	0.8491	C14—H14	0.9300
C5—C6	1.353 (5)	C20—C21	1.358 (6)
C5—H5	0.9300	C20—H20	0.9300
C2—C3	1.356 (5)	C15—H15	0.9300
C2—C1	1.389 (4)	C21—C22	1.387 (6)
C2—H2	0.9300	C21—H21	0.9300
C3—H3	0.9300	C22—H22	0.9300
C6—H6	0.9300	Cr1—O3	1.578 (3)
C10—C9	1.378 (5)	Cr1—O2	1.597 (3)
C10—H10	0.9300	Cr1—O1	1.614 (3)
C1—H1	0.9300		
			
Cr1—O4—Cr1^i^	130.3 (3)	N1—C1—H1	118.3
O1W^i^—Mg1—O1W	97.08 (17)	C2—C1—H1	118.3
O1W^i^—Mg1—N2	97.14 (10)	C8—C9—C10	119.2 (3)
O1W—Mg1—N2	94.70 (11)	C8—C9—H9	120.4
O1W^i^—Mg1—N2^i^	94.70 (11)	C10—C9—H9	120.4
O1W—Mg1—N2^i^	97.14 (10)	C9—C8—C7	120.1 (3)
N2—Mg1—N2^i^	162.09 (14)	C9—C8—H8	120.0
O1W^i^—Mg1—N1	88.13 (10)	C7—C8—H8	120.0
O1W—Mg1—N1	169.28 (11)	N3—C24—C16	122.5 (3)
N2—Mg1—N1	75.31 (9)	N3—C24—C23	118.2 (3)
N2^i^—Mg1—N1	91.73 (9)	C16—C24—C23	119.3 (3)
O1W^i^—Mg1—N1^i^	169.28 (10)	C15—C16—C24	116.8 (3)
O1W—Mg1—N1^i^	88.13 (10)	C15—C16—C17	123.7 (3)
N2—Mg1—N1^i^	91.73 (9)	C24—C16—C17	119.5 (3)
N2^i^—Mg1—N1^i^	75.31 (9)	N4—C23—C19	123.2 (3)
N1—Mg1—N1^i^	88.32 (13)	N4—C23—C24	117.8 (3)
N1—C12—C4	122.9 (2)	C19—C23—C24	119.0 (3)
N1—C12—C11	117.6 (2)	C20—C19—C23	117.7 (3)
C4—C12—C11	119.5 (2)	C20—C19—C18	122.6 (3)
C12—C4—C3	117.3 (3)	C23—C19—C18	119.7 (3)
C12—C4—C5	119.8 (3)	C13—N3—C24	117.6 (3)
C3—C4—C5	122.9 (3)	C22—N4—C23	116.3 (3)
C1—N1—C12	117.6 (2)	C17—C18—C19	121.0 (3)
C1—N1—Mg1	127.7 (2)	C17—C18—H18	119.5
C12—N1—Mg1	114.72 (17)	C19—C18—H18	119.5
C10—N2—C11	117.1 (3)	C18—C17—C16	121.4 (3)
C10—N2—Mg1	128.2 (2)	C18—C17—H17	119.3
C11—N2—Mg1	114.65 (18)	C16—C17—H17	119.3
C11—C7—C8	116.6 (3)	N3—C13—C14	124.2 (3)
C11—C7—C6	119.8 (3)	N3—C13—H13	117.9
C8—C7—C6	123.7 (3)	C14—C13—H13	117.9
N2—C11—C7	123.3 (2)	C15—C14—C13	118.5 (3)
N2—C11—C12	117.7 (2)	C15—C14—H14	120.7
C7—C11—C12	119.0 (3)	C13—C14—H14	120.7
Mg1—O1W—H1B	124.0	C21—C20—C19	119.2 (4)
Mg1—O1W—H1A	122.7	C21—C20—H20	120.4
H1B—O1W—H1A	101.3	C19—C20—H20	120.4
C6—C5—C4	120.5 (3)	C14—C15—C16	120.3 (3)
C6—C5—H5	119.8	C14—C15—H15	119.9
C4—C5—H5	119.8	C16—C15—H15	119.9
C3—C2—C1	119.4 (3)	C20—C21—C22	119.2 (4)
C3—C2—H2	120.3	C20—C21—H21	120.4
C1—C2—H2	120.3	C22—C21—H21	120.4
C2—C3—C4	119.4 (3)	N4—C22—C21	124.4 (4)
C2—C3—H3	120.3	N4—C22—H22	117.8
C4—C3—H3	120.3	C21—C22—H22	117.8
C5—C6—C7	121.4 (3)	O3—Cr1—O2	108.43 (17)
C5—C6—H6	119.3	O3—Cr1—O1	111.17 (19)
C7—C6—H6	119.3	O2—Cr1—O1	108.75 (16)
N2—C10—C9	123.7 (3)	O3—Cr1—O4	110.76 (14)
N2—C10—H10	118.1	O2—Cr1—O4	106.38 (17)
C9—C10—H10	118.1	O1—Cr1—O4	111.18 (18)
N1—C1—C2	123.4 (3)		

Symmetry codes: (i) −*x*+1, *y*, −*z*+1/2.

### Hydrogen-bond geometry (Å, °)

**Table:**

*D*—H···*A*	*D*—H	H···*A*	*D*···*A*	*D*—H···*A*
O1W—H1A···N3	0.85	2.08	2.876 (4)	156
O1W—H1B···O1	0.85	1.84	2.636 (4)	154
